# Development of 44 Novel Polymorphic SSR Markers for Determination of Shiitake Mushroom (*Lentinula edodes*) Cultivars

**DOI:** 10.3390/genes8040109

**Published:** 2017-03-24

**Authors:** Hwa-Yong Lee, Suyun Moon, Donghwan Shim, Chang Pyo Hong, Yi Lee, Chang-Duck Koo, Jong-Wook Chung, Hojin Ryu

**Affiliations:** 1 Department of Forest Science, Chungbuk National University, Cheongju 28644, Korea; hoasis82@chungbuk.ac.kr; 2 Department of Biology, Chungbuk National University, Cheongju 28644, Korea; sooym21@naver.com; 3 Department of Forest Genetic Resources, National Institute of Forest Science, Suwon 16631, Korea; shim104@korea.kr; 4 Theragen Etex Bio Institute, Suwon 16229, Korea; changpyo.hong@theragenetex.com; 5 Department of Industrial Plant Science and Technology, Chungbuk National University, Cheongju 28644, Korea; leeyi22@chungbuk.ac.kr

**Keywords:** shiitake mushroom, simple sequence repeat (SSR) marker, whole genome sequencing

## Abstract

The shiitake mushroom (*Lentinula* *edodes*) is one of the most popular edible mushrooms in the world and has attracted attention for its value in medicinal and pharmacological uses. With recent advanced research and techniques, the agricultural cultivation of the shiitake mushroom has been greatly increased, especially in East Asia. Additionally, demand for the development of new cultivars with good agricultural traits has been greatly enhanced, but the development processes are complicated and more challenging than for other edible mushrooms. In this study, we developed 44 novel polymorphic simple sequence repeat (SSR) markers for the determination of shiitake mushroom cultivars based on a whole genome sequencing database of *L. edodes*. These markers were found to be polymorphic and reliable when screened in 23 shiitake mushroom cultivars. For the 44 SSR markers developed in this study, the major allele frequency ranged from 0.13 to 0.94; the number of genotypes and number of alleles were each 2–11; the observed and expected heterozygosity were 0.00–1.00 and 0.10–0.90, respectively; and the polymorphic information content value ranged from 0.10 to 0.89. These new markers can be used for molecular breeding, the determination of cultivars, and other applications.

## 1. Introduction

The shiitake mushroom (*Lentinula edodes*) belongs to genus Lentinula, family Omphalotaceae, order Agaricales [1], and is a type of white rot fungi. This mushroom is commonly cultivated in Asian countries such as Korea, China, Japan, Taiwan, and others [2,3]. It constitutes approximately 17% of the global mushroom supply and is one of the most popular edible mushrooms in the world [4]. In addition to its value as a food, the shiitake mushroom is useful for pharmacological components such as lentinan, which shows antitumour activity [5,6].

To develop new mushroom cultivars, cross breeding, mutation breeding, transgenic breeding, and other approaches have been used [7]. The cultivar development of the shiitake mushroom is very difficult because the cultivation period is much longer than for the oyster mushroom (*Pleurotus ostreatus*), king oyster mushroom (*P*. *eryngii*), and winter mushroom (*Flammulina velutipes*) [8]. The traditional breeding for shiitake mushroom requires a lot of time and labor from strain selection for cultivation and identification of traits. Analysis of the genetic relationship between the relevant strains and the association of DNA markers in mushrooms can effectively increase breeding efficiency [9], because molecular markers save time in the selection process of the strain. During the last few decades, studies on the genetic diversity and population genetics in shiitake mushrooms have been conducted using various types of molecular markers, including restriction fragment length polymorphism (RFLP) [10], random amplified polymorphic DNA (RAPD) [11,12,13], amplified fragment length polymorphism (AFLP) [14], inter-simple sequence repeat (ISSR) [12,15,16], sequence-characterized amplified region (SCAR) [13,17,18], and sequence-related amplified polymorphism (SRAP) [12,16]. In spite of their diverse applications, the use of developed markers for the breeding and classification of shiitake mushrooms has been challengeable due to few available markers and little information regarding effectiveness for determination and specificity. Also, despite the advantages of simple sequence repeat (SSR) markers, such as co-dominant, highly polymorphic, reproducible, reliable, and distributed throughout the genome [19], the number of SSR markers available for Shiitake mushrooms are still scarce, with only a few expressed sequence tag-simple sequence repeat (EST-SSR) markers having been reported [20]. Thus, more reliable molecular markers are needed to enhance genetic analyses of the shiitake mushroom.

The traditional development of SSR markers was an experimentally long, labor-intensive, and economically costly process [21]. However, Next Generation Sequencing (NGS) technology is a powerful tool to find a large number of microsatellite loci through cost-effective and rapid identification [22]. In many recent studies, NGS-based transcriptome or genome sequencing is demonstrated to be efficient for the large-scale discovery of SSR loci in plants [21]. We have recently reported the genome sequence information of *L. edodes* [23], and have here developed higher polymorphic SSR markers based on the whole genome sequencing and determination of shiitake mushroom varieties. New SSR markers might be valuable tools to evaluate genetic variability and breeding in the shiitake mushroom.

## 2. Materials and Methods

### 2.1. Fungi Materials

To develop useful SSR markers in *L. edodes*, we selected the representative shiitake mushroom strains, which were successfully cultivated and distributed in the market in South Korea. Five strains from the National Institute of Forest Science in Korea Forest Service (http://www.forest.go.kr) and 18 strains from the Forest Mushroom Research Center (https://www.fmrc.or.kr/) were kindly provided. The list of strains are shown in Table 1. The mycelia of the strains were cultured for 10 days at 25 °C in darkness.

### 2.2. DNA Preparationument

For DNA extraction, the cultured mycelia were frozen in liquid nitrogen and ground into powders. DNA extraction was performed using a GenEX Plant Kit (Geneall, Seoul, Korea) following the manufacturer’s instructions. The extracted DNA was stored at −80 °C.

### 2.3. Discovery of SSR Markers

Over 1000 SSR loci of the shiitake mushroom were found in whole genome sequencing performed by Shim et al. [23] using *L. edodes* monokaryon strain B17 and comparing the resequencing data of 1 strain, Chamaram. We chose 205 SSR loci to test for polymorphism among the shiitake mushroom strains, and 44 SSR markers were finally selected for proper PCR conditions fixed in 23 strains (Supplementary Materials Figure S1).

The primer design parameters were set as follows: length range, 18–23 nucleotides with 21 as the optimum; PCR product size range, 150–200 bp; optimum annealing temperature (Ta), 58 °C; and GC content 50%–61%, with 51% as the optimum.

The extracted DNA for PCR templates was diluted to 20 ng/μL after checking concentration using a K5600 micro spectrophotometer (DaAn Gene, Guangzhou, China). The PCR reaction mixture consisted of 2 μL template DNA, 1 μL each of forward and reverse primer (5 pmol), 10 μL 2× i-Taq Master Mix (Intron biotechnology, Seongnam, Korea), and 6 μL distilled water. PCR reactions were performed as follows: 95 °C for 3 min; 35 cycles of 95 °C for 30 s, 58 °C for 30 s, and 72 °C for 30 s; and finally, 72 °C for 20 min. The size of the PCR product was confirmed by fragment analyzer (Advanced Analytical Technologies, Ankeny, IA, USA).

The amplified SSR loci were scored for 23 shiitake mushroom strains. Major allele frequency (M_AF_), number of genotypes (N_G_), number of alleles (N_A_), observed heterozygosity (H_O_), expected heterozygosity (H_E_), and polymorphic information content (PIC) values were calculated by using PowerMarker V3.25 [24].

## 3. Results and Discussion

The 44 SSR markers consist of di-, tri-, tetra-, and pentanucleotide DNA motifs. The SSR motifs used include 59.09% dinucleotide repeats, 31.82% trinucleotide repeats, 6.82% tetranucleotide repeats, and 2.27% pentanucleotide repeats. The SSR motifs are AG/GA, CT/TC, AT/TA, AC/CA, CG/GC, and TG dinucleotide repeats; AGG/AGA/GGA, CAG/CGA/GCA, AGA, GAT, GCT, GTT, TCA, and TCG trinucleotide repeats; TACT/TATC, and CTTT tetranucleotide repeats; and CTTCC pentanucleotide repeats (Table 2).

These 44 SSR markers were analyzed in 23 cultivars of shiitake mushroom. The major allele frequency (M_AF_) ranged from 0.13 to 0.94, with an average of 0.575. The number of genotypes (N_G_), ranged from 2 to 11, with an average of 5.5, and the number of alleles (N_A_) ranged from 2 to 11 with an average of 4.9 alleles. The observed heterozygosity (H_O_) ranged from 0.00 to 1.00, with an average of 0.309, and the expected heterozygosity (H_E_), ranged from 0.10 to 0.90, with an average of 0.552. The polymorphic information content (PIC) value ranged from 0.10 to 0.89, with an average of 0.511. M_AF_, N_G_, N_A_, H_O_, H_E_, and PIC per locus had wide ranges among the markers (Table 3).

SSR marker information for the determination of cultivars in other cultivated mushrooms have been released. In the SSR markers developed for the determination of black wood ear (*Auricularia* *auricular*-*judae*) cultivars, the PIC value ranged from 0.10 to 0.84, with an average of 0.47, and N_A_ ranged from 2–11, with an average of 4.7 (using 17 SSR markers and 16 cultivars) [25]. In the white button mushroom (*Agaricus* *bisporus*), the allele frequency ranged from 0.02–0.94, with an average of 0.18, and H_O_ ranged from 0.00 to 0.83, with an average of 0.35 (using 33 SSR markers, 6 cultivars and 17 wild types of *A*. *bisporus*, and 2 wild types of *A*. *bisporus* var. *burnettii*) [26]. In the golden needle mushroom (*F*. *velutipes*), the PIC value ranged from 0.13 to 0.69, with an average of 0.42 (using 55 SSR markers and 14 cultivars) [27]. In the oyster mushroom (*P*. *ostreatus*), the average of N_A_ was approximately 4.7. H_O_ ranged from 0.027 to 0.946, with an average of 0.398, and H_E_ ranged from 0.027 to 0.810, with an average of 0.549 (using 36 SSR markers, and 37 cultivars including *P*. *sajor*-*caju*, 1 *P*. *eryngii*, 1 *P*. *cornucopiae* var. *citrinopileatus*, and 1 *P*. *nebrodensis*) [28]. The SSR markers developed in this study showed similar diversity values with the SSR markers of other edible mushrooms. The 20 markers with PIC values above 0.6 in the 44 newly developed markers are useful for identification among strains or cultivars of shiitake mushroom. The SSR markers developed in this study were able to distinguish 23 shiitake mushroom strains, which are broadly cultivated in Korea (Supplementary Materials Figure S2).

## 4. Conclusions

We have developed 44 novel SSR markers for the determination of shiitake mushroom cultivars. SSR marker development was performed based on NGS-based genome sequencing data. The efficacy and availability of the developed SSR markers were evaluated by application to distinguishing 23 shiitake mushroom cultivars. These new markers can be used for molecular breeding, cultivar determination, genetic structure research, and further applications in cultivated and wild types of shiitake mushrooms.

## Figures and Tables

**Table 1:** List of shiitake mushroom (*Lentinula edodes*) strains.

No.	Strain Name	Cultivar Name
1	KFRI 623	Baekhwahyang
2	KFRI 174	Soohyangko
3	KFRI 551	Poongnyunko
4	KFRI 2924	Sanmaru 1 h_O_
5	KFRI 2925	Sanmaru 2 h_O_
6	SJ101	Sanjo 101 h_O_
7	SJ102	Sanjo 102 h_O_
8	SJ103	Sanjo 103 h_O_
9	SJ108	Sanjo 108 h_O_
10	SJ109	Sanjo 109 h_O_
11	SJ110	Sanjo 110 h_O_
12	SJ111	Sanjo 111 h_O_
13	SJ301	Sanjo 301 h_O_
14	SJ501	Sanjo 501 h_O_
15	SJ702	Sanjo 702 h_O_
16	SJ704	Sanjo 704 h_O_
17	SJ705	Sanjo 705 h_O_
18	SJ706	Sanjo 706 h_O_
19	SJ707	Sanjo 707 h_O_
20	SJ708	Sanjo 708 h_O_
21	SJ709	Sanjo 709 h_O_
22	SJ710	Sanjo 710 h_O_
23	SJCAR	Chamaram

No. 1~5: National Institute of Forest Science in Korea Forest Service; No. 6~23: Forest Mushroom Research Center.

**Table 2:** Characteristics of the 44 simple sequence repeat (SSR) markers for shiitake mushroom (*Lentinula edodes*). Ta, annealing temperature.

Marker	Primer Sequences (5′–3′)	Expected Size	Motif	GenBank Accession No.	Ta (°C)	Description
RL-LE-017	F: GTGCACTGTGCGATTGTTC	199	CA	NM-0418-000001	59	Subtilase family, Pro-kumamolisin, activation domain
	R: CAGCAAGGATGACTCTTGGA					
RL-LE-018	F: CCCACAGGTTTACAGAGTTCCT	152	TA	NM-0418-000002	59	-
	R: GTGGACATCCACCTTTTGTC					
RL-LE-019	F: TACTTTCGAAGCCAGCCA	191	CTTCC	NM-0418-000003	58	-
	R: GTAGCTCTTTAGGTCTGCTTGG					
RL-LE-020	F: GACGGAGTTGTCAAGATCTACC	173	AT	NM-0418-000004	58	-
	R: ACCTAGGCTTTGCTCTACACAG					
RL-LE-021	F: GCTTGAAGAGCGAGTTTGAG	200	AG	NM-0418-000005	58	Uso1/p115-like vesicle tethering protein, head region
	R: CAAGACACGCTTCGTAGTCA					
RL-LE-022	F: CAAACGAAGGAGGAGGTAGTTC	199	GCA	NM-0418-000006	60	-
	R: GAGTCCATTACTCATCGTGCTG					
RL-LE-023	F: GAGGTAGCACCAGTTGAGGTAA	150	AGA	NM-0418-000007	59	-
	R: ATAAGACTTCGTCTCGTCCTGC					
RL-LE-024	F: GTAAGGCTTTAGGACTCGTCG	187	TC	NM-0418-000008	59	-
	R: CCACAGATGTTTCCGAGTTG					
RL-LE-025	F: TTGGGAGATGCGAGTAGTTC	200	AT	NM-0418-000009	58	PCI domain, 26S proteasome subunit RPN7
	R: ATTCAGTCGCTCAGTAGGAGAC					
RL-LE-026	F: GATTTGACGCTCACATCCC	197	AG	NM-0418-000010	59	-
	R: CCCCTAAGTATGAGCTTCCGTA					
RL-LE-027	F: GGGTCACAAGAGCAATGTAGAC	192	CT	NM-0418-000011	59	-
	R: CTGTATGGTGATCAAGGACGAG					
RL-LE-028	F: GAGACGACACGAGGAATTTG	174	CA	NM-0418-000012	59	Ras family
	R: GTCGTTCTCATTGGAGACTCTG					
RL-LE-029	F: CAAGATCCGTCGCCATATAC	178	GGA	NM-0418-000013	58	-
	R: AACTCACCCTCGTCTACCTCTAC					
RL-LE-030	F: CTTGGGAAGGAGGAATGG	164	TACT	NM-0418-000014	59	-
	R: GTGGGACCAATATGAGGACAGT					
RL-LE-031	F: ACTTCAGTTACAGCGACTCTGC	194	CAG	NM-0418-000015	58	PAS domain, PAS domain
	R: GTCGGAGACTGTGCGTTC					
RL-LE-032	F: GTAGAAGGTGCACCAGTTTCTG	190	AGG	NM-0418-000016	59	-
	R: CGTCTCTTACCAGGAATCACAC					
RL-LE-033	F: GACAGAAGAAGGACTTACCAGC	197	CT	NM-0418-000017	58	-
	R: CCAGAGCCCAAGGATAACTT					
RL-LE-034	F: AGGTGGAGTTGAGTGTTTGAGG	170	TA	NM-0418-000018	59	-
	R: AGTCTCAGGAGACCTTCACTAGC					
RL-LE-035	F: GTCGGAAGCTTTATGACACG	196	GAG	NM-0418-000019	58	-
	R: TCAACTTTCTGCTCCCTCAC					
RL-LE-036	F: TCTAGCTCGGTGAGCAATGT	181	CG	NM-0418-000020	59	-
	R: GAGACCTTGAGGAAGAGACTCC					
RL-LE-037	F: CTCTCATCCTTAAGAACCTCCC	198	CGA	NM-0418-000021	59	-
	R: GAGAAGCTTACATATGGTCCCG					
RL-LE-038	F: CGTTTGAGTGTCAACGGTCT	199	AT	NM-0418-000022	59	-
	R: CATGTCAGACTAGTCAGGGGTC					
RL-LE-039	F: GTACGAGGACAGCAATACAGC	200	GA	NM-0418-000023	58	-
	R: GCTTCTATATCTCCTCTGCCCT					
RL-LE-040	F: GGTTTCCTCTCACACCTTACCT	178	CT	NM-0418-000024	59	-
	R: GAAAATGTGCTGTAGCGAGC					
RL-LE-041	F: GGTGTATAAAGAGAGCCCTTGG	153	AG	NM-0418-000025	59	SNF2 family N-terminal domain, Ring finger domain
	R: CCCCTTATCCAGTCTACTGCTAC					
RL-LE-042	F: TCCTCTGCTTCACTAAGTCTCC	167	TCG	NM-0418-000026	58	STAG domain
	R: AGTACTCGCAAGGCAGGTAAG					
RL-LE-043	F: GTTCGTCACTCGGTACTTTCC	177	AC	NM-0418-000027	58	-
	R: AGATGCAGGAGTATGACCTGAC					
RL-LE-044	F: GTAAGCCTAAGGAGGGTGGAG	198	GGA	NM-0418-000028	59	WH1 domain, P21-Rho-binding domain
	R: CACCTCCTTCATCTGGTCC					
RL-LE-045	F: ACATCTGAGAGGTCGTACGCT	164	CA	NM-0418-000029	59	Cytochrome b5-like heme/steroid binding domain, Acyl-CoA dehydrogenase, C-terminal domain
	R: GTACCGAAGCGAGCAAGTT					
RL-LE-046	F: GCACGCAGTGATGAATAGAGAG	154	AG	NM-0418-000030	60	Cytochrome P450
	R: ACACTTACGGATTTGGCAGG					
RL-LE-047	F: CTACCACTCGTCACTCCTTAGGT	194	TC	NM-0418-000031	60	-
	R: GAAGGAGTGTGAAGCTGAAACC					
RL-LE-048	F: GTGGTGAAGTTACCGACAGG	197	GC	NM-0418-000032	58	Pectate lyase
	R: AGGTGCCCAACTTCTGGT					
RL-LE-049	F: GCTACCTAGATCCTCCTAGATCG	184	GA	NM-0418-000033	58	-
	R: GACTACGTCAAGTTGAGGATGC					
RL-LE-050	F: TACCCGAAGGAACTAACGAGTC	200	TG	NM-0418-000034	59	-
	R: GTCGTCGTATAACGACTCATCC					
RL-LE-051	F: ACTCTGCTGCCACTCTTGAC	172	CT	NM-0418-000035	58	Short chain dehydrogenase
	R: GACCGTCTCTAGCTTCTTGATG					
RL-LE-052	F: CTAAAGCAACGGTAGACGTAGG	178	GCT	NM-0418-000036	58	-
	R: ACAACAAACGCTAGAGCGAG					
RL-LE-053	F: CTCAACGTCTCATTCCCTTC	179	GTT	NM-0418-000037	58	-
	R: CTCGAGTTGAGGGTGAGGTTAT					
RL-LE-054	F: GAATCAGCTAGACCATCTCTGC	200	GAT	NM-0418-000038	58	-
	R: TCTTTACCCGTCTTGTCTGC					
RL-LE-055	F: CTGGGGATAGTGATATCGAGAG	165	CTTT	NM-0418-000039	58	-
	R: GTAAACCCGCTCCTTTGTGT					
RL-LE-056	F: GCGGTCCTGAGTACAAAGTAGT	159	TATC	NM-0418-000040	58	-
	R: CTACGTACGGAGGAATCTAGTGC					
RL-LE-057	F: AGGAGAACGGAACCGAAGTTAC	160	AT	NM-0418-000041	59	Protein of unknown function DUF262
	R: CAGTAGACGTTGCTTACTGCAC					
RL-LE-058	F: GTCGTAGAACTTGCACGAGTC	163	GCA	NM-0418-000042	57	-
	R: GAAGTTCTCCGCTATCCTCTC					
RL-LE-059	F: CGGAGATGTACCAATTCCTG	193	TG	NM-0418-000043	59	-
	R: GCATTCGCCGTCTATACGAT					
RL-LE-060	F: ACTCAGCGCACATCTAGCTT	191	TCA	NM-0418-000044	58	-
	R: CAGGGAGAAGAAAGTCACGA					

**Table 3:** Diversity statistics from each primer used for screening 23 cultivars of shiitake mushroom (*Lentinula edodes*).

Marker	M_AF_	N_G_	N_A_	H_O_	H_E_	PIC
RL-LE-017	0.13	11	11	0.00	0.9	0.89
RL-LE-018	0.29	6	6	0.08	0.77	0.74
RL-LE-019	0.41	7	5	0.87	0.68	0.63
RL-LE-020	0.67	3	3	0.00	0.5	0.45
RL-LE-021	0.52	11	10	0.43	0.68	0.66
RL-LE-022	0.70	5	4	0.04	0.48	0.45
RL-LE-023	0.63	6	5	0.09	0.55	0.51
RL-LE-024	0.80	4	4	0.13	0.33	0.31
RL-LE-025	0.43	7	6	0.64	0.72	0.68
RL-LE-026	0.34	9	6	0.68	0.74	0.69
RL-LE-027	0.50	5	5	0.26	0.64	0.58
RL-LE-028	0.37	6	4	0.35	0.73	0.68
RL-LE-029	0.81	3	3	0.00	0.32	0.29
RL-LE-030	0.65	4	3	0.26	0.51	0.46
RL-LE-031	0.46	4	6	1.00	0.68	0.63
RL-LE-032	0.87	3	4	0.22	0.24	0.22
RL-LE-033	0.46	10	9	0.48	0.72	0.69
RL-LE-034	0.50	6	6	0.22	0.68	0.64
RL-LE-035	0.65	5	5	0.17	0.52	0.47
RL-LE-036	0.72	3	3	0.04	0.42	0.35
RL-LE-037	0.50	6	4	0.39	0.62	0.55
RL-LE-038	0.39	7	9	1.00	0.72	0.68
RL-LE-039	0.50	3	3	0.13	0.56	0.46
RL-LE-040	0.52	6	5	0.52	0.66	0.61
RL-LE-041	0.43	4	5	0.09	0.65	0.58
RL-LE-042	0.80	4	3	0.04	0.33	0.3
RL-LE-043	0.87	2	2	0.00	0.23	0.2
RL-LE-044	0.76	3	3	0.42	0.37	0.32
RL-LE-045	0.39	6	5	0.35	0.73	0.69
RL-LE-046	0.43	7	6	0.09	0.71	0.66
RL-LE-047	0.48	10	6	0.35	0.69	0.65
RL-LE-048	0.43	8	5	0.39	0.7	0.66
RL-LE-049	0.41	4	5	1.00	0.66	0.6
RL-LE-050	0.94	2	2	0.00	0.1	0.1
RL-LE-051	0.28	9	6	0.65	0.79	0.76
RL-LE-052	0.70	5	5	0.09	0.47	0.43
RL-LE-053	0.39	10	10	0.61	0.79	0.77
RL-LE-054	0.76	5	3	0.09	0.39	0.36
RL-LE-055	0.72	4	4	0.13	0.44	0.38
RL-LE-056	0.59	5	4	0.68	0.58	0.53
RL-LE-057	0.87	3	3	0.09	0.23	0.22
RL-LE-058	0.93	3	3	0.04	0.12	0.12
RL-LE-059	0.35	6	5	0.30	0.74	0.69
RL-LE-060	0.91	2	2	0.17	0.16	0.15
Mean	0.575	5.5	4.9	0.309	0.552	0.511

(M_AF_), Major allele frequency; (N_G_), number of genotypes; (N_A_), number of alleles; (H_O_), observed heterozygosity; (H_E_), expected heterozygosity; and (PIC), polymorphic information content.

## Supplementary Materials

The following are available online at www.mdpi.com/2073-4425/8/4/109/s1, Figure S1: The development process of SSR marker developed using genome sequencing for *Lentinula edodes*, Figure S2: Distinguished *Lentinula edodes* strains using the 44 novel SSR markers developed in this study.
