# Supplementary material for: Development of 44 Novel Polymorphic SSR Markers for Determination of Shiitake Mushroom (Lentinula edodes) Cultivars
Source: Genes (Basel). 2017 Mar 24;8(4):109. doi: 10.3390/genes8040109 (PMC5406856; doi:10.3390/genes8040109)
Supplement: Supplementary file 1 [file genes-08-00109-s001.pdf]

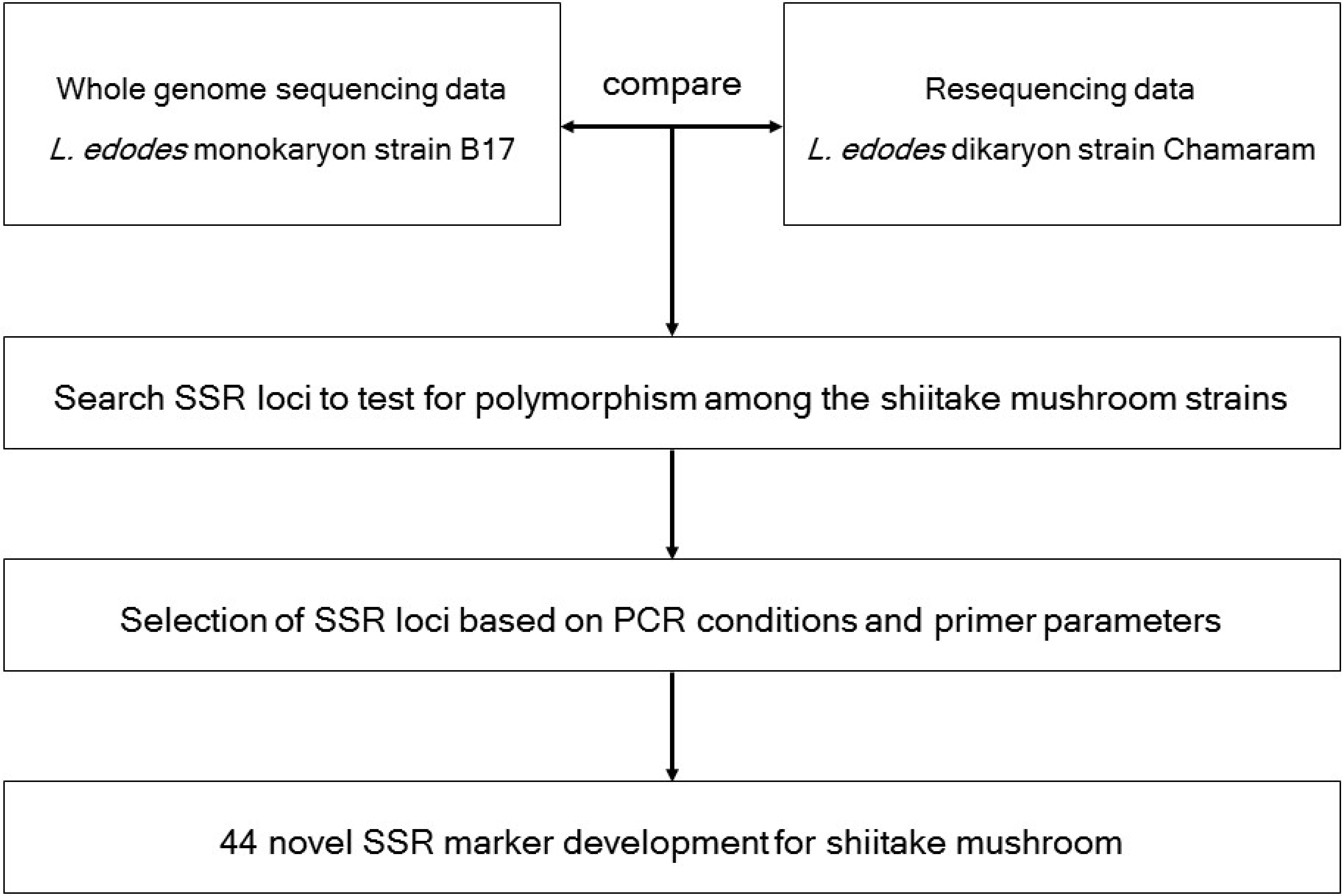

Figure S1. The development process of SSR marker developed using genome sequencing for *Lentinula edodes*.

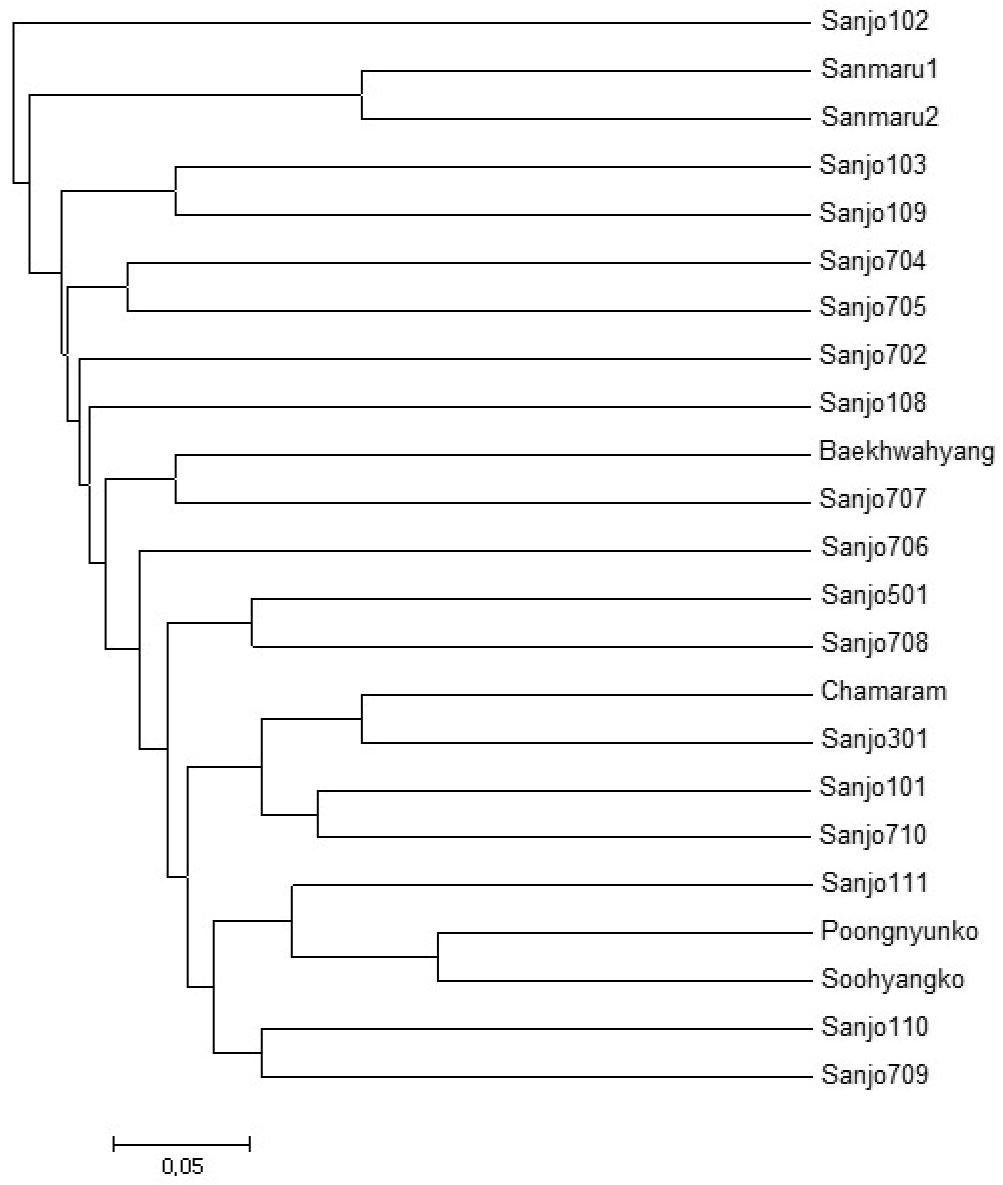

Figure S2. Distinguished *Lentinula edodes* strains using the 44 novel SSR markers developed in this study.
